# The clinical application of partial removal periodontal surgery in the therapy of epulis

**DOI:** 10.1097/MD.0000000000016107

**Published:** 2019-07-05

**Authors:** Yaqiao Zhu, Huihui Zhang, Chengzhang Li

**Affiliations:** a The State Key Laboratory Breeding Base of Basic Science of Stomatology (Hubei-MOST) & Key Laboratory of Oral Biomedicine Ministry of Education; b Department of Periodontology, School and Hospital of Stomatology, Wuhan University, Wuhan, Hubei Province; c The Affiliated Huizhou Stomatologic Hospital of Medical College of Jinan University & Huizhou Stomatologic Hospital, Huizhou, Guangdong Province, P. R. China.

**Keywords:** fibrous epulis, keratinized gingiva, partial removal periodontal surgery

## Abstract

**Background:**

The aim of this study was to compare the clinical effects between traditional surgery and minimally invasive periodontal surgery in the treatment of epulis.

**Methods:**

A total of 33 cases of patients diagnosed with fibrous epulis were randomly divided into traditional surgery group and minimally invasive periodontal surgery group. After the different procedures, several parameters were detected to evaluate the effects of minimally invasive periodontal surgery.

**Results:**

Preoperative bleeding index and plaque index, adopt rank, and test showed no significant differences between the 2 groups. After 12 weeks, gingival papilla filling index in experiment group is statistically higher than control group, and shows the statistical differences (*P* < .05). The width of keratinized gingiva in experiment group grew more than that in control group, and showed the statistical differences (4.68 ± 0.30 vs 3.00 ± 0.28 mm, *P* < .05). No recurrence of fibrous epulis was found during the subsequent 6 months to 2 years follow-up after the surgeries.

**Conclusion:**

Minimally invasive periodontal surgery that reserved tumor epithelium could have a better effect than the traditional surgery in the selected patients.

## 1 Introduction

Epulis, with a tumor-like appearance, is a kind of localized hyperplasia commonly seen in the gingiva. Some studies have indicated that this disease could be caused by low-grade local irritation, traumatic injury, hormonal factors, or some drugs.^[1–4]^ It has reported 4 cases of epulis arising from cyclosporine in patients with chronic graft versus host disease.^[5,6]^ In addition, epulis are most likely to occur at the age of 20 years old in women, because the hormone levels of women, such as such as estrogen and progesterone, are easily changed at this stage.^[7]^

The methods to treat epulis depend on its size and position and excisional biopsy is the recommended method for most epulis. However, 2 disadvantages need to be concerned. First, postoperative recurrence should not be ignored. Usually, incomplete resection can lead to the failure to remove local stimulation factors and secondary injury in the surgical area, which will stimulate the tissue hyperplasia.^[2,3]^ To prevent the postoperative recurrence, radical initial therapy can be used to remove the local microstimulation factors in preoperative phase. Moreover, improving surgical skills leads to complete surgical treatment. Moreover, strict medical orders can contribute to avoiding secondary injury in the surgical area. Second, soft tissue deformity in anterior areas will cause aesthetic defects and the periodontist could not prevent the soft tissue defect. Previous studies reported that tissue engineering could reconstruct human oral mucosa to treat soft-tissue defect after surgery.^[8]^ The retainment of epulis mucosa during the excisional biopsy might be a potential method to reconstruct the normal shape. However, the clinical application still needs to be investigated.

In this study, we tried to explore a new procedure named partial removal periodontal surgery to treat the epulis. The aim of this improved treatment was to avoid soft tissue defect by maximized retention of soft tissue.

## 2 Methods and procedures

### 2.1 Patients

A prospective study was performed in our center between October 2015 and September 2016. The inclusive criteria include diagnosed as fibrous epulis; tooth mobility was less then degree I; without a history of familial gingival hyperplasia. The exclusion criteria include moderate and severe periodontitis; history of pregnancy; taking immunosuppressive agents or calcium channel antagonists; and with a history of familial gingival hyperplasia. Informed written consent and ethics review board approval was obtained.

### 2.2 Surgical procedures

**Figure 1:**
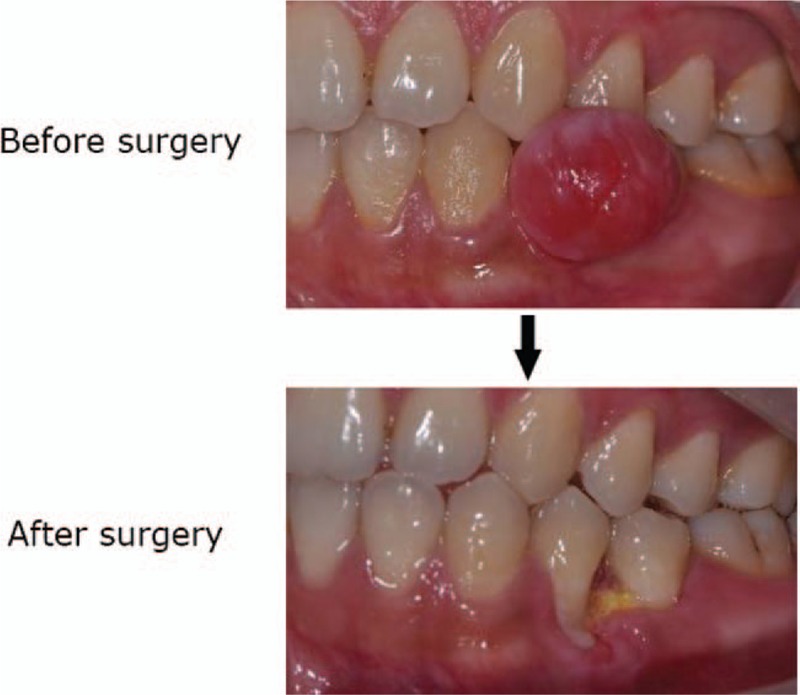
The representative image of traditional surgical procedure before and after surgery.

**Figure 2:**
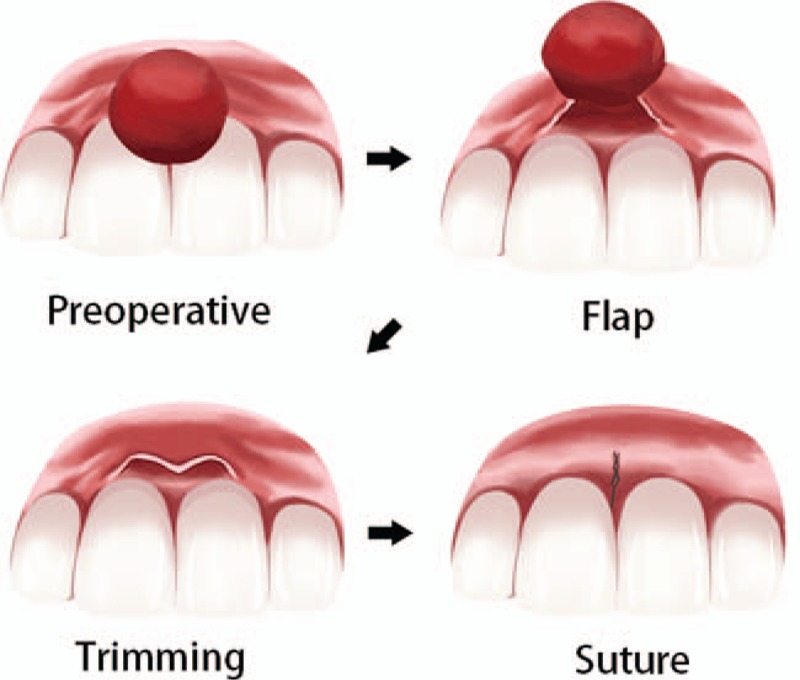
The sketch map of our new surgical procedure.

The surgical procedures were divided into the traditional surgery group and minimally invasive periodontal surgery group. In the traditional surgery group, gingival tumor lesions and surrounding 1 mm normal tissues were excised. Therefore, more soft tissue defects could be found from these patients (Fig. 1). In the minimally invasive periodontal surgery group, the tumor tissues were excised and the epithelial and connective tissues of the tumor were retained. The thickness of the retained tissues was about 0.5 mm. The reserved epithelial tissues and connective tissues were then covered the wound surface (Fig. 2).

### 2.3 Clinical parameters and statistical analysis

The postoperative pain index was analyzed by visual analogue scale (VAS). Bleeding index (BI), plaque index (PLI), gingival recession (GR), and papilla filled index (PFI) were also calculated. Statistical Package for Social Sciences version 16.0 (SPSS, Chicago, IL) was used for all statistical analysis and *t* test and Fisher exact test were used. The *P* values <.05 were considered statistically significant.

## 3 Results

A total of 33 patients including 13 men and 20 women were eligible to participate in this study and then divided into traditional surgery group (n = 15) and minimally invasive periodontal surgery group (n = 18). Age (39.7 ± 1.9 vs 35.3 ± 3.2, *P* > .05) and tumor size (10.5 ± 1.1 vs 11.4 ± 0.9 mm, *P* > .05) showed no significant differences between the 2 groups. The BI and PLI were calculated before the operations and the results showed that there were no significant differences between the 2 groups for these 2 indexes (*P* > .05) (Table 1).

**Table 1:**
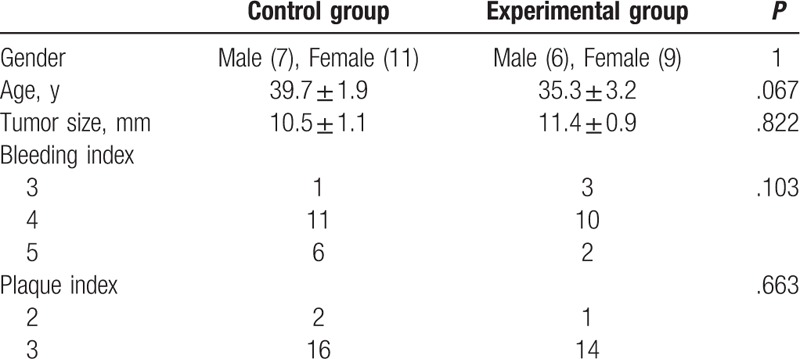
The characters of patients included in this study.

After we finished the operations, we recorded the postoperative pain scores and the VAS results showed that the patients of minimally invasive periodontal surgery group suffered a milder pain than traditional surgery group (*P* < .05) (Table 2). Next, gingival retraction (GR) and PFI were also calculated for 2 groups at the 12 weeks after the operations. The results demonstrated that the minimally invasive periodontal surgery could significantly improve GR (2.69 ± 0.48 vs 0.77 ± 0.16 mm, *P* < .05), PFI (*P* < .05), and variation of keratinized gingival width (1.08 ± 0.13 vs 3.00 ± 0.28 mm, *P* < .05) (Table 3). In addition, postoperative satisfaction was also analyzed and minimally invasive periodontal surgery group showed a better result. At last, a 6 months to 2 years follow-up showed that no recurrence was found in all the patients (Table 4).

**Table 2:**
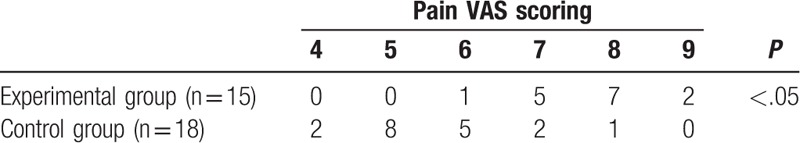
The comparison of postoperative pain VAS scoring between experimental group and control group after the surgery.

**Table 3:**
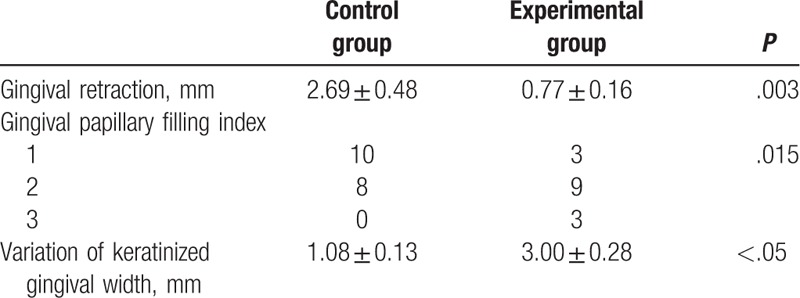
The comparisons of three clinical parameters between experimental group and control group after the surgery.

**Table 4:**
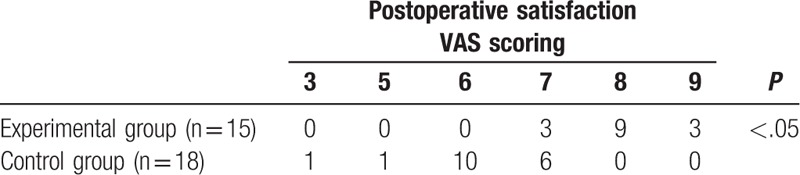
The comparison of postoperative satisfaction VAS scoring between experimental group and control group after the surgery.

## 4 Discussion

Epulis is a localized chronic inflammatory hyperplasia of gingiva, which comes from connective tissue of periodontal ligament and alveolar process. Traditional surgical excision of epulis can lead to soft tissue defects. The histopathological features of the epithelia of fibrous epulis are the continuous inflammation of the connective tissue under the epithelia and the separation of the acanthosis cells from the basal layer cells.^[9,10]^ Therefore, the different histopathological types for the epithelia and the tumor make it possible to retain the epithelial. In this manuscript, we retained the epithelial and subcutaneous connective tissue of the tumor to produce a suitable cover after the operation and then compared the short-term and long-term therapeutic effects with the traditional method.

Our results indicated that the postoperative pain of the experimental group was significantly less than that of the control group and the difference between the 2 groups was statistically significant. In traditional surgery group, it was necessary to remove the proper normal tissue around the tumor and severe tissue congestion and edema could be caused after the operation. Irritants could easily stimulate the wound and cause pain. Because of the soft tissue defect, the healing of the tissue began with the formation of the surface blood clot. It could cover and protect the wound. The epithelial cells began to crawl from the edge of the wound to the wound 12 to 24 hours after the operation and then form thin skin epithelium to cover the wound for about 2 weeks. The keratinization of the epithelium could be completed for about 4 to 5 weeks after the operation.^[11,12]^ On the contrary, minimally invasive surgery only removed the lesions of the tumor and the granulation tissue. However, the epithelium and the upper subcutaneous part of connective tissue were retained. The tissue flap was closely sutured after the operation and the stimulation of the intratumoral excision could be avoided. After the operation, the healing period of the experimental group was shorter because of the close coverage of soft tissue. Therefore, the pain of the experimental group was lighter than that of the control group. Moreover, the retention of soft tissue in the gingival tumor area could also be compared by using GR and PFI. As a result, postoperative satisfaction was relatively higher in the experimental group.

It is reported that the recurrence rate after conventional resection is 16%, and reoperation is usually needed after recurrence.^[13]^ The main causes of recurrence are incomplete resection, local irritation residuals, or mechanical damage to the operative area. However, in this study, the cases of gingival tumor resection in the control group and the minimally invasive resection of the gingival tumor in the experimental group were followed for 0.5 to 2 years and no recurrent cases were found. The reasons were all the cases were treated with complete periodontal nonsurgical treatment before operation to eliminate local irritation factors; all the periodontal membrane and proper bone dressing should be completely removed during the operation; and oral hygiene education was performed after the operation to ensure the patient's oral hygiene. Therefore, no recurrent cases were found in both the control group and the experimental group.

To sum, minimally invasive periodontal surgery acquired a better effect compared with the traditional surgery from our study. This method could be used as a potential option for selective patients with epulis. More samples should be included to validate our hypothesis in the future.

## Author contributions

**Conceptualization:** Yaqiao Zhu.

**Formal analysis:** Huihui Zhang, Chengzhang Li.

**Investigation:** Chengzhang Li.

**Writing – original draft:** Yaqiao Zhu.

**Writing – review & editing:** Yaqiao Zhu.

## References

[1] Kamal R Dahiya P Puri A Oral pyogenic granuloma: various concepts of etiopathogenesis. J Oral Maxillofac Pathol 2012;16:79–82. 22434943 10.4103/0973-029X.92978 PMC3303528

[2] Neville BW Damm DD Allen CM Bouquot JE Oral and Maxillofacial Pathology. 3rd edn. St. Louis, MO: Saunders-Elsevier; 2009.

[3] Mussalli NG Hopps RM Johnson NW Oral pyogenic granuloma as a complication of pregnancy and the use of hormonal contraceptives. Int J Gynaecol Obstet 1976;14:187–91. 10213 10.1002/j.1879-3479.1976.tb00592.x PMC8333992

[4] Punde PA Malik SA Malik NA Idiopathic huge pyogenic granuloma in young and old: an unusually large lesion in two cases. J Oral Maxillofac Pathol 2013;17:463–6. 24574675 10.4103/0973-029X.125222 PMC3927358

[5] Piccin A Tagnin M Vecchiato C Graft-versus-host disease (GvHD) of the tongue and of the oral cavity: a large retrospective study. Int J Hematol 2018;108:615–21. 30144000 10.1007/s12185-018-2520-5

[6] Suh JD Blackwell KE Nabili V Graft-versus-host disease of the tongue. Otolaryngol Head Neck Surg 2009;140:272–3. 19201305 10.1016/j.otohns.2008.10.015

[7] Jafarzadeh H Sanatkhani M Mohtasham N Oral pyogenic granuloma: a review. J Oral Sci 2006;48:167–75. 17220613 10.2334/josnusd.48.167

[8] Izumi K Neiva RF Feinberg SE Intraoral grafting of tissue-engineered human oral mucosa. Int J Oral Maxillofac Implants 2013;28:e295–303. 24066347 10.11607/jomi.te11 PMC4193471

[9] Cutright DE The histopathologic findings in 583 cases of epulis fissuratum. Oral Surg Oral Med Oral Pathol 1974;37:401–11. 4591192 10.1016/0030-4220(74)90113-3

[10] Desai D Pandith S Jeergal PA Fibroblastic variant of osteosarcoma: a challenge in diagnosis & management. Open Dent J 2010;4:211–7. 21243071 10.2174/1874210601004010211 PMC3020523

[11] Eversole LR Clinical Outline of Oral Pathology: Diagnosis and Treatment. 3rd edn. Hamilton, Ontario, Canada: BC Decker Inc; 2002.

[12] Warnakulasuriya S Burket's oral medicine: diagnosis and treatment. Br Dent J 2003;194:579–1579.

[13] Qiu X Dong Z Zhang J Lobular capillary hemangioma of the tracheobronchial tree: a case report and literature review. Medicine (Baltimore) 2016;95:e5499. 27902613 10.1097/MD.0000000000005499 PMC5134768

